# A Comparison of Health-Related Quality of Life in Patients with Periprosthetic Joint Infection, Patients with Fracture-Related Infections and the General Population—A Multicenter Analysis of 384 Patients from the Section “Musculoskeletal Infections” of the German Society for Orthopaedics and Traumatology

**DOI:** 10.3390/jcm14217649

**Published:** 2025-10-28

**Authors:** Yves Gramlich, Nike Walter, Jasper Frese, Eva Simone Steinhausen, Mathias Holz, Igor Lazic, Mario Morgenstern, Björn Schaper, Sascha Gravius, Jobst Hansberg, Dominik Gruszka, Martin Clauss, Matthias Schnetz, Rita Schoop, Sabrina Janoud, Benjamin Schlossmacher, Jan-Hendrik Christensen, Sebastian Meller, Volker Alt

**Affiliations:** 1 BG Unfallklinik Frankfurt am Main, Department of Trauma Surgery and Orthopaedic Surgery, 60389 Frankfurt am Main, Germany; 2 Agaplesion Markus Krankenhaus, 60389 Frankfurt am Main, Germany; 3 Department of Trauma Surgery, University Hospital Regensburg, 93053 Regensburg, Germany; 4 Zentrum Klinische Forschung-zkf, BG Klinikum Hamburg, 21033 Hamburg, Germany; 5 Department of Trauma, Hand and Reconstructive Surgery, University Hospital Essen, University of Duisburg-Essen, 45147 Essen, Germany; 6 Department of Orthopedic and Trauma Surgery, BG Klinikum Duisburg, 47249 Duisburg, Germany; 7 Department of Trauma Surgery, University Hospital Schleswig-Holstein, 23538 Lübeck, Germany; 8 Department of Orthopaedics and Sports Orthopaedics, Technical University of Munich, 81675 Munich, Germany; 9 Department of Orthopaedic Surgery and Traumatology, University Hospital Basel, 4031 Basel, Switzerland; 10 Center for Musculoskeletal Infections (ZMSI), University Hospital Basel, 4031 Basel, Switzerland; 11 BG Klinik Ludwigshafen, 67071 Ludwigshafen am Rhein, Germany; 12 Department of Orthopaedic and Trauma Surgery, University Medical Centre Mannheim, 68167 Mannheim, Germany; 13 Department of Hip Arthropasty und Periimplant Infections, Center of Musculoskeletal Surgery, Charité University Medicine Berlin, 10117 Berlin, Germany; 14 Septische Unfallchirurgie und Orthopädie, BG Klinikum Hamburg, 21033 Hamburg, Germany; 15 Regensburger University Center for Musculoskeletal Infections (RUCMI), University Hospital Regensburg, 93053 Regensburg, Germany

**Keywords:** quality of life, periprosthetic joint infection, fracture-related infection

## Abstract

**Background:** Periprosthetic joint infections (PJIs) and fracture-related infections (FRIs) are severe complications in orthopedic and trauma surgery. This study aimed to evaluate patient-reported health-related quality of life (HRQoL) in patients treated for PJI and FRI across multiple centers in Germany and Switzerland. **Methods:** A retrospective cohort study was conducted in nine hospitals based on a project of the Section “Musculoskeletal Infections” of the German Society of Orthopaedics and Traumatology. Patients treated in 2021 were included to ensure a minimum 12-month follow-up. Diagnoses were verified using EBJIS and FRI consensus criteria. HRQoL was assessed via telephone interviews using the EQ-5D questionnaire and a visual analog scale (VAS). Reinfection rates and additional treatments were also recorded. Generalized estimating equations (GEEs) with age and sex as covariates and clustering on center were used to compare groups, with *p*-values adjusted for multiple testing using the Benjamini–Hochberg false discovery rate (FDR). **Results:** In total, 384 patients were included (197 PJI, 187 FRI). Compared with the German reference population, both groups reported markedly reduced HRQoL across all EQ-5D domains. After adjustment, PJI patients had higher odds of reporting problems in self-care (OR 1.69, 95% CI 1.13–2.54, FDR-*p* = 0.033), usual activities (OR 1.68, 95% CI 1.14–2.47, FDR-*p* = 0.033), and pain/discomfort (OR 2.35, 95% CI 1.31–4.21, FDR-*p* = 0.033) compared with FRI patients. VAS scores were similar between groups (PJI: 52.8, FRI: 55.5; *p* = 0.489). Reinfection was associated with significantly worse outcomes: in FRI, usual activities were more impaired (OR 2.41, 95% CI 1.56–3.72, FDR-*p* = 0.0004); in PJI, reinfection was linked to worse mobility (OR 2.14, 95% CI 1.55–2.95, FDR-*p* < 0.001), self-care (OR 3.70, 95% CI 2.49–5.49, FDR-*p* < 0.001), and usual activities (OR 3.92, 95% CI 2.76–5.57, FDR-*p* < 0.001). **Conclusion:** This multicenter study highlights the burden of PJI and FRI on patient-reported outcomes with a significant reduction in quality of life compared to the standard population. PJI patients, in particular, experienced greater impairments in mobility, self-care, and usual activities. Reinfection was associated with poorer outcomes, underscoring the importance of patient-centered rehabilitation in managing musculoskeletal infections.

## 1. Introduction

Bone and joint infections, particularly periprosthetic joint infections (PJIs) and fracture-related infections (FRIs), represent serious complications in orthopedic and trauma surgery. These infections not only pose significant challenges in terms of treatment but also have a profound impact on patients’ long-term functional outcomes and overall quality of life [1,2]. PJI usually occurs after joint replacement surgery and often necessitates multiple revision procedures, prolonged antibiotic therapy, and, in some cases, permanent joint dysfunction. Similarly, FRI can develop after surgical fixation procedures, leading to delayed healing, reoperations, and substantial disability with varying clinical appearances [3]. Given the increasing number of orthopedic procedures and fracture fixations performed globally, understanding the long-term consequences of these infections is essential [4,5].

Health-related quality of life (HRQoL) is a key outcome measure that reflects the overall well-being of patients following medical interventions. While the clinical management of PJI and FRI has been extensively studied, and research has highlighted that higher postoperative psychological burden is associated with reduced outcomes and increased infection risk in primary arthroplasty [6,7], less attention has been given to the impact of these infections on patients’ daily lives, functional independence, and psychological well-being [8]. Previous studies have suggested that chronic infections may lead to persistent pain, mobility restrictions, and emotional distress [9,10,11,12], yet comprehensive multicenter data on long-term HRQoL in this patient population remain limited. Therefore, a detailed assessment of patient-reported outcomes is essential to guide future treatment strategies and rehabilitation programs.

The primary aim of this study is to evaluate the long-term HRQoL outcomes in patients treated for PJI and FRI in multiple healthcare centers in Germany and Switzerland. Specifically, this study seeks to (1) assess HRQoL from patients with PJI and FRI using standardized patient-reported outcome measures, including the EQ-5D questionnaire and visual analog scale (VAS), (2) compare the HRQoL outcomes of PJI and FRI patients with age-matched reference data from the German general population, and (3) assess the impact of reinfection versus primary infection on patient-reported HRQoL outcomes.

## 2. Materials and Methods

This study included patients treated for PJI or FRI in nine different hospitals in Germany and Switzerland and was conducted as a project from the Section “Musculoskeletal Infections” of the German Society for Orthopaedics and Traumatology (Deutsche Gesellschaft für Orthopädie und Unfallchirurgie (DGOU). This study was conducted in accordance with the Declaration of Helsinki. Ethical approval was obtained from the following ethics committees: University Hospital Schleswig-Holstein, Kiel (D 598/23, 3 November 2023), BG Clinic Ludwigshafen (No. 2023-17398, 19 January 2024), BG Trauma Center Frankfurt, Ethics Committee of the State Medical Association of Hessen (2023-3585-zvBO, 30 November 2023), Ethics Committee Northwest and Central Switzerland (EKNZ; Project-ID 2024-01244, Basel, 10 October 2024), University of Duisburg-Essen (24-11808-BO, 15 April 2024), University Hospital Regensburg (23-3468-101, 31 August 2023), Ethics Committee of the University of Lübeck (2024-166, 19 February 2024), Technical University of Munich (714/20 S, 15 March 2024), Ethics Committee of Charité Berlin (EA4/114/23, 10 August 2024).

The indication classification was retrospectively verified through medical records, applying the European Bone and Joint Infection Society (EBJIS) and FRI consensus definition criteria to ensure standardized and objective diagnosis confirmation [13,14]. All patients received treatment in 2021 ensuring a minimum follow-up period of 12 months at the time of data collection in the years 2023 and 2024. Health-related quality of life was assessed only once in a cross-sectionally manner, via telephone interview at least 12 months after treatment. Patients were asked about any signs of reinfection, additional surgical interventions required, and their current health status. For the latter, the European Quality of Life 5 Dimensions (EQ-5D-3L) questionnaire was applied [15].

This instrument assesses five key dimensions related to different functional domains: mobility, self-care, daily activities, pain/discomfort, and anxiety/depression. Additionally, the visual analog scale (VAS) methodology was used, and scores were assessed via telephone. To provide context for the health-related quality of life outcomes, patient responses were compared with German reference population values to assess deviations from expected health status in the general population [16]. The reference data were age-matched. For the subgroup analysis of infection recurrence, the “reinfection” group was defined as patients with either (i) a documented history of previous infection in the same anatomical region, or (ii) treatment failure during follow-up, i.e., relapsed infection requiring additional surgical treatment.

### Statistical Analysis

Data analysis was conducted using SPSS Statistics version 28.0 (IBM, Armonk, NY, USA). Descriptive statistics are presented as mean ± standard deviation (SD) for continuous variables and absolute frequencies (n) and percentages (%) for categorical variables. Unadjusted comparisons of categorical variables (e.g., sex distribution) were performed using the chi-square test for independence; if expected cell counts were <5, Fisher’s exact test was applied.

Each EQ-5D-3L domain was modeled as a binary outcome (any problem vs. no problems; levels 2–3 vs. level 1). Between-group effects (PJI vs. FRI) and within-group effects (reinfection vs. primary) were estimated using generalized estimating equations (GEE) with a binomial family and exchangeable working correlation, clustering on center to account for the multicenter design. All models included group (exposure), age (continuous), and sex as covariates.

Continuous variables (age and VAS score) were assessed for equality of variances using Levene’s test. Given the difference in variance between groups, Welch’s *t*-test, a robust variation of the independent *t*-test, was used for group comparisons of continuous variables. Additionally, Cohen’s d effect size was calculated to assess the magnitude of the difference, with interpretation based on standard thresholds: small (d = 0.2), medium (d = 0.5), and large (d ≥ 0.8) [17]. All comparisons across EQ-5D domains and subgroups were adjusted for multiple testing using the Benjamini–Hochberg false discovery rate (FDR) procedure. A *p*-value of <0.05 after adjustment was considered statistically significant.

## 3. Results

The total study cohort included 384 patients, with 197 in the PJI group and 187 in the FRI group (Table 1 and Table 2). In the PJI group, the most commonly affected anatomical locations were the hip (n = 107, 54.3%), followed by the knee (n = 79, 40.1%) and shoulder (n = 11, 5.6%). A total of 92 patients (46.7%) presented with a reinfection, indicating that they had previously experienced a periprosthetic joint infection before the current episode. 36 patients of the 197 PJI patients (18.3%) experienced treatment failure, requiring additional surgical intervention due to persistent or recurrent infection. In the FRI group, 57 patients (30.5%) presented with a reinfection, indicating prior infection episodes in the same anatomical region. In the complete cohort, 42 cases (22.4%) were classified as open fractures. In total, 48 patients (25.7%) required additional surgery due to reinfection-related treatment failure, while 139 patients (74.3%) achieved successful fracture consolidation without further interventions. The analysis of demographic characteristics revealed no statistically significant difference in sex distribution between the PJI (female: 43.2%, male: 56.8%) and FRI cohort (female: 36.9%, male: 63.1%) group (*p* = 0.462, chi-square test). However, a significant difference was observed in age, with the PJI group being significantly older (mean age: 70.1 ± 11.1 years) compared to the FRI group (58.0 ± 15.0 years), (*p* < 0.001, Welch’s *t*-test).

### 3.1. HRQoL in PJI and FRI Patients

**Figure 1:**
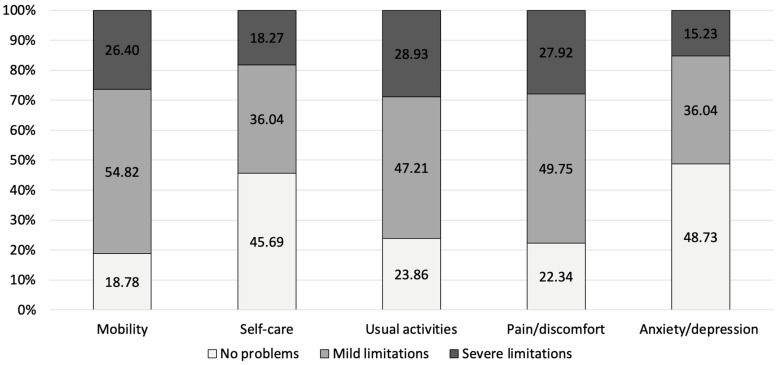
Scores of the subdimensions of the EQ-5D reported by PJI patients.

**Figure 2:**
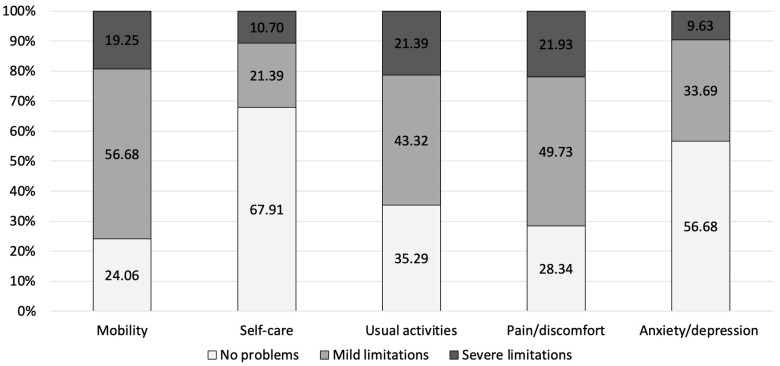
Scores of the subdimensions of the EQ-5D reported by FRI patients.

For PJI, the highest proportion of severe limitations was observed in mobility (26.4%), followed by usual activities (28.93%) and pain/discomfort (27.92%). The anxiety/depression dimension had the largest proportion of participants without limitations (48.73%) and the lowest proportion with severe limitations (15.23%) (Figure 1). For FRI, the highest overall limitation burden (i.e., the sum of mild and severe limitations) was observed in the mobility domain, with 75.93% of respondents reporting either mild (56.68%) or severe (19.25%) limitations. Similarly, high levels of limitations were reported for pain/discomfort (71.66%) and usual activities (64.71%) (Figure 2).

### 3.2. Comparison of HRQoL Outcomes Between PJI and FRI Patients with Age-Matched Reference Data from the German General Population

**Figure 3:**
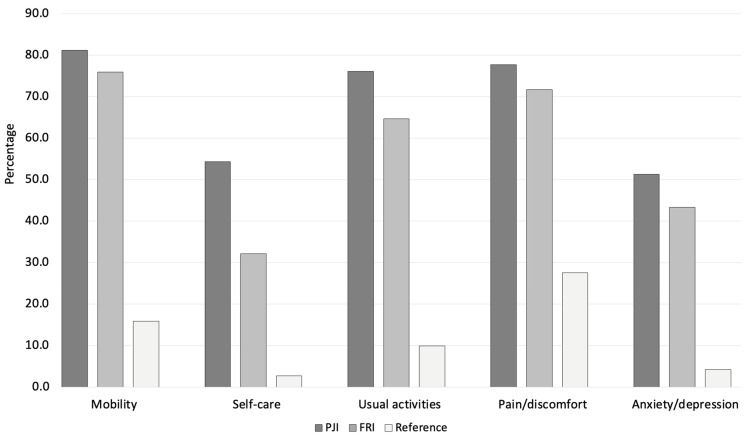
Proportions of patients reporting any limitations in each EQ-5D subdomain for PJI (shown in dark grey), FRI (shown in light grey) and a German reference population (shown in white). All comparisons were statistically significant on a *p* < 0.001 level.

When comparing both patient cohorts to the German reference population, PJI and FRI patients showed significantly higher impairment in all EQ-5D dimensions (*p* < 0.01 for all comparisons). In the mobility domain, limitations were reported by 81.2% of PJI and 75.9% of FRI patients, while only 15.9% of the general population experienced similar restrictions. Self-care difficulties were noted in 54.3% of PJI and 32.1% of FRI patients, compared to just 2.7% in the reference population. Impairments in usual activities were reported by 76.1% of PJI and 64.7% of FRI patients, whereas only 9.9% of the general population experienced comparable limitations. Similarly, pain or discomfort was present in 77.7% of PJI and 71.7% of FRI patients, while 27.6% of the general population reported similar symptoms. Anxiety or depression was reported by 51.3% of PJI and 43.3% of FRI patients, significantly higher than the 4.3% observed in the German reference population (Figure 1, Figure 2 and Figure 3). Compared to age-matched German VAS score reference values of the general population (68.6 ± 1.1 for PJI and 72.9 ± 1.0 for FRI), the effect size for the difference was large for both comparisons, with PJI patients showing a Cohen’s d of −0.90 and FRI patients a Cohen’s d of −0.89, indicating a substantial reduction in perceived health status.

### 3.3. Comparison of HRQoL Between Patients with PJI and FRI

After adjustment for age, sex, multicenter clustering, and multiple testing (FDR), PJI patients had significantly higher odds of reporting problems in self-care (OR 1.69, 95% CI 1.13–2.54, FDR-*p* = 0.033), usual activities (OR 1.68, 95% CI 1.14–2.47, FDR-*p* = 0.033), and pain/discomfort (OR 2.35, 95% CI 1.31–4.21, FDR-*p* = 0.033) compared with FRI patients. Differences in mobility (OR 1.71, 95% CI 0.88–3.34, FDR-*p* = 0.100) and anxiety/depression (OR 1.54, 95% CI 0.91–2.61, FDR-*p* = 0.099) were not statistically significant after FDR correction. The mean VAS score was 52.8 ± 24.8 in the PJI group and 55.5 ± 27.5 in the FRI group, with no significant difference between groups (*p* = 0.489).

### 3.4. Impact of Recurrence of Infection After Index Treatment Versus Primary Infection on Patient-Reported HRQoL Outcomes

**Figure 4:**
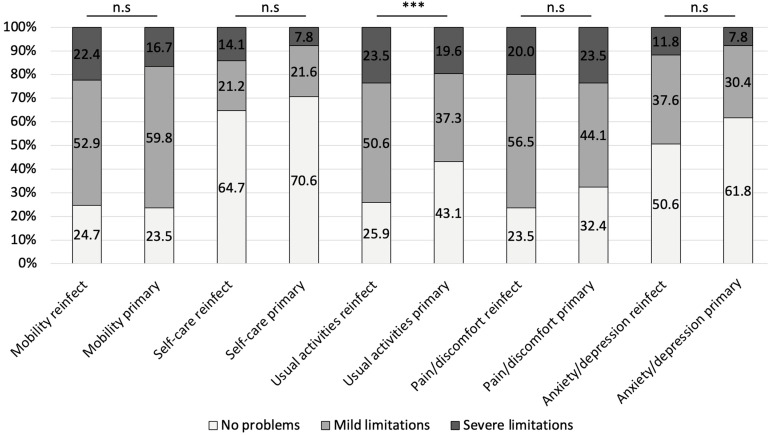
Proportions of patients reporting no problems, mild limitations, and severe limitations in each EQ-5D subdomain for FRI reinfection and primary FRI cases. Statistical comparisons were performed using generalized estimating equations (GEE) with binomial distribution, adjusted for age and sex and clustered by center. Reported *p*-values are Benjamini–Hochberg false discovery rate (FDR) adjusted. *** FDR-*p* < 0.001.

**Figure 5:**
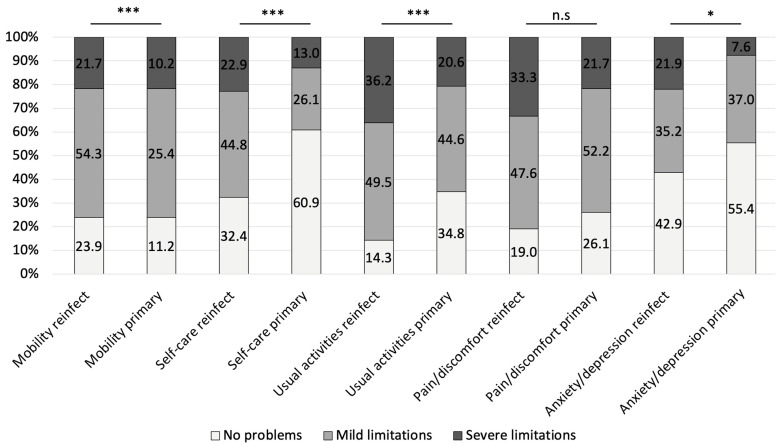
Proportions of patients reporting no problems, mild limitations, and severe limitations in each EQ-5D subdomain for PJI reinfection and primary PJI cases. Statistical comparisons were performed using generalized estimating equations (GEE) with binomial distribution, adjusted for age and sex and clustered by center. Reported significance levels are based on Benjamini–Hochberg false discovery rate (FDR) adjusted *p*-values. *** FDR-*p* < 0.001, * FDR-*p* < 0.05.

After adjustment for age, sex, multicenter clustering, and multiple testing (FDR), the comparison between FRI reinfection and primary FRI cases showed a significant difference only in the domain of usual activities (OR 2.41, 95% CI 1.56–3.72, FDR-*p* < 0.001), with reinfected patients reporting greater limitations. No significant differences were observed in mobility, self-care, pain/discomfort, or anxiety/depression (Figure 4). Within the PJI group, reinfection was associated with markedly higher odds of limitations in mobility (OR 2.14, 95% CI 1.55–2.95, FDR-*p* < 0.001), self-care (OR 3.70, 95% CI 2.49–5.49, FDR-*p* < 0.001), usual activities (OR 3.92, 95% CI 2.76–5.57, FDR-*p* < 0.001) and anxiety/depression (OR 1.87, 95% CI 1.01–3.47, FDR-*p* = 0.046). Differences in pain/discomfort (FDR-*p* = 0.059) did not remain statistically significant after correction (Figure 5).

## 4. Discussion

This multicenter study provides a comprehensive analysis of HRQoL in patients treated for PJI and FRI, demonstrating significant impairments across multiple domains. Compared to age-matched reference populations, both PJI and FRI patients reported substantially lower HRQoL scores, highlighting the long-term burden of bone and joint infections. Our findings indicate increased anxiety and depression scores among reinfected FRI patients, which aligns with prior studies demonstrating higher rates of depression and anxiety in FRI patients postoperatively. Notably, prior studies have shown that FRI patients experience a higher psychological burden following reinfection, with increasing anxiety levels after surgery and lower expectations of returning to normal health [18]. Additionally, prevalence of depressive symptoms was shown to be up to four times higher in PJI patients undergoing two-stage exchange procedures compared to those undergoing aseptic revisions (40.5% vs. 10.8%, *p* < 0.01) [19]. Thus, implementing routine psychological screening in orthopedic patient populations and incorporating mental health professionals in the management may represent a valuable future direction [20,21]. Our findings support these trends, suggesting that reinfection exacerbates psychological distress and contributes to a lower perceived quality of life. Our study identified significant impairments in mobility, self-care, and usual activities among PJI patients, as well as in usual activities in FRI patients. These findings align with prior studies reporting increased rates of assistive device usage among PJI patients, with an adjusted OR of 3.10 for total hip arthroplasty (THA) patients and 5.40 for total knee arthroplasty (TKA) patients [22]. Furthermore, it was shown that compared to primary THA patients, PJI patients exhibit lower Oxford Hip Scores and a greater likelihood of requiring assisted living (21% vs. 12%, OR 2.0, *p* = 0.01) [23]. These results are consistent with our findings that mobility impairments remain one of the most persistent challenges in both PJI and FRI patients. Similarly, previous studies have shown that PJI patients undergoing two-stage revision surgeries experience lasting functional impairments, with a substantial proportion unable to negotiate stairs (20.5%), suffering from worse physical health (39.6%), and reporting worse mental health (25.2%) [24]. Our data support this, emphasizing the need for intensive post-treatment rehabilitation strategies. Encouragingly, a prospective study over a 12-month period has shown the potential for improvements in mobility, self-care, and usual activities suggesting that many patients regain functional independence, although a subset continues to experience significant challenges [25]. Also, longitudinal FRI studies have reported notable functional recovery, with severe mobility limitations decreasing from 20.0% preoperatively to 0% at 12 months [26]. However, this was not reflected in our cohort, where functional impairments remained persistent, particularly in reinfected cases, suggesting that differences in patient selection, treatment protocols, and reinfection rates may play a significant role in long-term outcomes. In line, prior studies have also shown permanent disability in up to 64% (n = 111) patients after FRI treatment [27] and it has been reported that FRI patients generally exhibit worse HRQoL outcomes compared to non-FRI patients [28] and the general population [29]. This underscores the importance of a multidisciplinary treatment approach, which has been shown to enhance patient outcomes [30,31].

### Limitations

Despite the strengths of this study, several limitations should be acknowledged. First, the data was obtained cross-sectional, meaning that no preoperative baseline is available.

Further, the retrospective nature of data collection may introduce recall bias, particularly in patient-reported outcomes obtained through telephone interviews. Second, the reliance on self-reported HRQoL measures, while validated and widely used, may be subject to subjective interpretation and variability between respondents. Factors such as psychological resilience, social support, and pre-existing health conditions could influence how individuals perceive their quality of life, potentially leading to response bias. Additionally, while we used standardized instruments like the EQ-5D and VAS, these tools may not fully capture all dimensions of post-infection recovery, such as fatigue, physical endurance, and cognitive impact. Other potentially relevant influencing factors, including side effects of prolonged antibiotic therapy, the need for mobility aids and dependency on professional or family care, were not systematically captured in this retrospective design. Another limitation is the lack of direct comparisons between different surgical treatment strategies. While surgical management of PJI and FRI varies widely, ranging from debridement and implant retention (DAIR) to two-stage revisions and amputation, our study did not specifically evaluate the impact of different treatment choices on HRQoL outcomes. Detailed surgical information, such as the use of spacers or specific revision procedures, was not consistently available across centers and therefore could not be analyzed. Additionally, while our study adjusted for reinfection status, we did not account for potential differences in infection severity, microbial resistance profiles, or systemic comorbidities, all of which may have influenced outcomes. It has been previously reported that patients with ‘complex’ or ‘limited treatment options’ PJI experience significantly lower EQ-5D scores compared to ‘uncomplicated’ cases [26] suggesting that there are more key factors to classify the complexity of bone and joint infection that warrant further investigation [3]. Due to the heterogeneity of patient populations across centers and the limited sample size within subgroups, the statistical power for further stratified analyses was restricted, and the results should be interpreted as exploratory. Finally, while the multicenter nature of this study improves its generalizability, our cohort was limited to hospitals in Germany and Switzerland, and the findings may not be fully applicable to other healthcare settings with different infection management protocols, rehabilitation access, or socioeconomic factors.

## 5. Conclusions

In conclusion, our findings contribute to the growing body of evidence highlighting the long-term burden of bone and joint infections on patient-reported quality of life. Compared to prior studies, our cohort demonstrated persistently lower HRQoL scores, particularly in patients with recurrent PJI, supporting the notion that reinfection leads to greater physical disability. Given the high rates of mobility impairments, self-care limitations, and restricted usual activities observed in our study, future research should focus on developing patient-centered rehabilitation programs, and multidisciplinary care pathways to improve long-term outcomes.

## Figures and Tables

**Table 1:** Demographics of PJI patients.

Variable	PJI
Total number of patients	197
Mean age in years	70.1 ± 11.1
Sex (female/male)	85/112
Anatomical localization	
Hip	107 (54.3%)
Knee	79 (40.1%)
Shoulder	11 (5.6%)
Patients with prior infection	92 (46.7%)
Number of surgeries for index treatment	1.8 (range 1–8)
Recurrence of infection	36 (18.3%)

**Table 2:** Demographics of FRI patients.

Variable	FRI
Total number of patients	187
Mean age in years	58.0 ± 15.0
Sex (female/male)	69/118
Anatomical localization	
Femur	23 (12.3%)
Acetabulum	3 (1.6%)
Tibia	94 (50.3%)
Fibula	6 (3.2%)
Ankle	22 (11.8%)
Foot	13 (7.0%)
Ulna	2 (1.1%)
Radius	5 (2.7%)
Patients with prior infection	57 (30.5%)
Number of surgeries for index treatment	2.5 (range 1–16)
Recurrence of infection	48 (25.7%)

## Data Availability

The datasets generated and/or analyzed during the current study are available from the corresponding author upon reasonable request.

## References

[1] Nelson S.B., Pinkney J.A., Chen A.F., Tande A.J. (2023). Periprosthetic Joint Infection: Current Clinical Challenges. Clin. Infect. Dis..

[2] Moriarty T.F., Metsemakers W.-J., Morgenstern M., Hofstee M.I., Vallejo Diaz A., Cassat J.E., Wildemann B., Depypere M., Schwarz E.M., Richards R.G. (2022). Fracture-Related Infection. Nat. Rev. Dis. Primer.

[3] Alt V., McNally M., Wouthuyzen-Bakker M., Metsemakers W.-J., Marais L., Zalavras C., Morgenstern M. (2024). The FRI Classification—A New Classification of Fracture-Related Infections. Injury.

[4] Harris E., Clement N., MacLullich A., Farrow L. (2024). The Impact of an Ageing Population on Future Increases in Hip Fracture Burden: Insights from the Scottish Hip Fracture Audit. Bone Jt. J..

[5] Shichman I., Roof M., Askew N., Nherera L., Rozell J.C., Seyler T.M., Schwarzkopf R. (2023). Projections and Epidemiology of Primary Hip and Knee Arthroplasty in Medicare Patients to 2040–2060. JB JS Open Access.

[6] Pan X., Wang J., Lin Z., Dai W., Shi Z. (2019). Depression and Anxiety Are Risk Factors for Postoperative Pain-Related Symptoms and Complications in Patients Undergoing Primary Total Knee Arthroplasty in the United States. J. Arthroplast..

[7] Harmer J.R., Wyles C.C., Duong S.Q., Morgan Iii R.J., Maradit-Kremers H., Abdel M.P. (2023). Depression and Anxiety Are Associated with an Increased Risk of Infection, Revision, and Reoperation Following Total Hip or Knee Arthroplasty. Bone Jt. J..

[8] Lueck E., Schlaepfer T.E., Schildberg F.A., Randau T.M., Hischebeth G.T., Jaenisch M., Ossendorff R., Wirtz D.C., Wimmer M.D. (2022). The Psychological Burden of a Two-Stage Exchange of Infected Total Hip and Knee Arthroplasties. J. Health Psychol..

[9] Wimalan B., Rupp M., Alt V., Walter N. (2023). The Patients’ Perspective—A Qualitative Analysis of Experiencing a Fracture-Related Infection. Front. Psychol..

[10] Aichmair A., Pastl D., Frank B.J.H., Simon S., Mitterer J.A., Dominkus M., Hofstaetter J.G. (2024). High Demand for Psychological Support in Patients Who Have Periprosthetic Hip and Knee Joint Infections: An Analysis of 13,976 Patients. J. Arthroplast..

[11] Knebel C., Menzemer J., Pohlig F., Herschbach P., Burgkart R., Obermeier A., Von Eisenhart-Rothe R., Mühlhofer H.M.L. (2020). Peri-Prosthetic Joint Infection of the Knee Causes High Levels of Psychosocial Distress: A Prospective Cohort Study. Surg. Infect..

[12] Walter N., Loew T., Hinterberger T., Alt V., Rupp M. (2024). Managing More than Bones: The Psychological Impact of a Recurrent Fracture-Related Infection. Bone Jt. Open.

[13] McNally M., Sousa R., Wouthuyzen-Bakker M., Chen A.F., Soriano A., Vogely H.C., Clauss M., Higuera C.A., Trebše R. (2021). The EBJIS Definition of Periprosthetic Joint Infection: A Practical Guide for Clinicians. Bone Jt. J..

[14] Metsemakers W.J., Morgenstern M., McNally M.A., Moriarty T.F., McFadyen I., Scarborough M., Athanasou N.A., Ochsner P.E., Kuehl R., Raschke M. (2018). Fracture-Related Infection: A Consensus on Definition from an International Expert Group. Injury.

[15] Devlin N., Parkin D., Janssen B. (2020). An Introduction to EQ-5D Instruments and Their Applications. Methods for Analysing and Reporting EQ-5D Data.

[16] Szende A., Janssen B., Cabases J. (2014). Self-Reported Population Health: An International Perspective Based on EQ-5D.

[17] Cohen J. (2013). Statistical Power Analysis for the Behavioral Sciences.

[18] Hegde V., Bracey D.N., Johnson R.M., Dennis D.A., Jennings J.M. (2022). Increased Prevalence of Depressive Symptoms in Patients Undergoing Revision for Periprosthetic Joint Infection. Arthroplast. Today.

[19] Walter N., Rupp M., Baertl S., Hinterberger T., Alt V. (2022). Prevalence of Psychological Comorbidities in Bone Infection. J. Psychosom. Res..

[20] Krenn V.T., Bönigk M.S., Trampuz A., Liebisch M., Perka C., Meller S. (2025). Coping with Chronic Periprosthetic Joint Infection after Failed Revision of Total Knee and Hip Arthroplasty: A Qualitative Study on Patient’s Experiences in Treatment and Healing. PLoS ONE.

[21] Mur I., Jordán M., Rivera A., Pomar V., González J.C., López-Contreras J., Crusi X., Navarro F., Gurguí M., Benito N. (2020). Do Prosthetic Joint Infections Worsen the Functional Ambulatory Outcome of Patients with Joint Replacements? A Retrospective Matched Cohort Study. Antibiotics.

[22] Wildeman P., Rolfson O., Söderquist B., Wretenberg P., Lindgren V. (2021). What Are the Long-Term Outcomes of Mortality, Quality of Life, and Hip Function after Prosthetic Joint Infection of the Hip? A 10-Year Follow-up from Sweden. Clin. Orthop..

[23] Shichman I., Sobba W., Beaton G., Polisetty T., Nguyen H.B., Dipane M.V., Hayes E., Aggarwal V.K., Sassoon A.A., Chen A.F. (2023). The Effect of Prosthetic Joint Infection on Work Status and Quality of Life: A Multicenter, International Study. J. Arthroplast..

[24] Walter N., Mohokum M., Loew T., Rupp M., Alt V. (2024). Healing beyond the Joint: Addressing Mental Health in Periprosthetic Joint Infection in a Prospective Longitudinal Study. J. Psychosom. Res..

[25] Walter N., Loew T., Hinterberger T., Mohokum M., Alt V., Rupp M. (2025). Mental Health Implications of Fracture-Related Infections: A Longitudinal Quality of Life Study. Bone Jt. Res..

[26] Khalili P., Tevell S., Fischer P., Hailer N.P., Wolf O. (2023). Analysis of Fracture-Related Infections from Swedish Insurance Claims between 2011 and 2021. Sci. Rep..

[27] Buijs M.A.S., Haidari S., IJpma F.F.A., Hietbrink F., Govaert G.A.M. (2024). What Can They Expect? Decreased Quality of Life and Increased Postoperative Complication Rate in Patients with a Fracture-Related Infection. Injury.

[28] Walter N., Rupp M., Hierl K., Pfeifer C., Kerschbaum M., Hinterberger T., Alt V. (2021). Long-Term Patient-Related Quality of Life after Fracture-Related Infections of the Long Bones. Bone Jt. Res..

[29] Rupp M., Walter N., Popp D., Hitzenbichler F., Heyd R., Geis S., Kandulski M., Thurn S., Betz T., Brochhausen C. (2023). Multidisciplinary Treatment of Fracture-Related Infection Has a Positive Impact on Clinical Outcome—A Retrospective Case Control Study at a Tertiary Referral Center. Antibiotics.

[30] Carlson V.R., Dekeyser G.J., Certain L., Pupaibool J., Gililland J.M., Anderson L.A. (2020). Clinical Experience with a Coordinated Multidisciplinary Approach to Treating Prosthetic Joint Infection. Arthroplast. Today.

[31] Hotchen A.J., Wismayer M.G., Robertson-Waters E., McDonnell S.M., Kendrick B., Taylor A., Alvand A., McNally M. (2021). The Joint-Specific BACH Classification: A Predictor of Outcome in Prosthetic Joint Infection. eClinicalMedicine.

